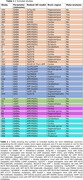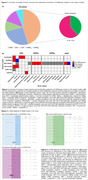# A systematic review with meta‐analysis of the GABAergic system in transgenic rodent models of amyloidosis and tauopathy

**DOI:** 10.1002/alz.092415

**Published:** 2025-01-03

**Authors:** André Nunes Mensch, Giovanna Carello‐Collar, Vanessa G. Ramos, Bruna Bellaver, Pamela C.L. Ferreira, Pedro Rosa‐Neto, Tharick A. Pascoal, Marco Antônio de Bastiani, Eduardo R. Zimmer

**Affiliations:** ^1^ Universidade Federal do Rio Grande do Sul, Porto Alegre, Rio Grande do Sul Brazil; ^2^ Universidade Federal do Rio Grande do Sul, Porto Alegre, RS Brazil; ^3^ University of Pittsburgh, Pittsburgh, PA USA; ^4^ Department of Psychiatry, University of Pittsburgh School of Medicine, Pittsburgh, PA USA; ^5^ Department of Neurology and Neurosurgery, McGill University, Montréal, QC Canada; ^6^ Department of Psychiatry and Neurology, Pittsburgh, PA USA; ^7^ Brain Institute of Rio Grande do Sul ‐ Pontifícia Universidade Católica do Rio Grande do Sul, Porto Alegre, Rio Grande do Sul Brazil

## Abstract

**Background:**

Alzheimer’s disease (AD) is characterized by the accumulation of amyloid‐β (Aβ) plaques and tau tangles in the brain, and neurotransmission dysfunctions. Indeed, our group recently demonstrated that the γ‐aminobutyric acid (GABA)ergic system is vulnerable to AD pathology in humans. However, whether this vulnerability is also present in AD rodent models is still unknown. Thus, we aimed to examine the GABAergic system in rodent models of amyloidosis and tauopathy.

**Method:**

We systematically reviewed the literature following the PRISMA 2020 Statement. We searched PubMed and Web of Science from database inception to March 2023 for studies reporting GABA, glutamate decarboxylase (GAD) 65/67, GABA transporters (GAT), and GABA_A_ and GABA_B_ receptors in the brain of 3xTg‐AD, 5xFAD, APP/PSEN1, McGill‐R‐Thy1‐APP, Tg2576, TauPS2APP, TgCRND8, APP SweDI, and hAPP‐J20 AD rodent models. Then, we performed a random‐effects meta‐analysis of standardized mean differences (SMD) using the metafor and metaviz packages in R (FDR‐adjusted p‐value < 0.05).

**Result:**

The search identified 3,631 articles and 27 met the inclusion criteria (Table 1 and Figure 1A). The systematic review showed decreased levels of GAD65/67 in the hippocampus, dentate gyrus, and hilus of TauPS2APP, hippocampus of TgCRND8, hippocampus and cortex of APP SweDI, and suprachiasmatic nucleus of Tg2576 mouse models. However, no differences in the cortex and cerebellum of APP/PSEN1 and hippocampus of hAPP‐J20 mice were found (Figure 1B). APP/PSEN1 had decreased vGAT in the hippocampus and increased in cortex (Figure 1B). GABA_A_ receptors (Figure 1B) were decreased in APP/PSEN1 mice, while GABA_B_ receptors (Figure 1B) remained unchanged. Due to insufficient number of studies, we meta‐analyzed only GABA. There were no differences in GABA levels in the cortex of APP/PSEN1 and Tg2576 mouse models (Figures 2A and 2B, p = 0.226 and 0.968, respectively), and in the hippocampal GABA levels in 5xFAD mice (Figure 2C, p = 0.924).

**Conclusion:**

Here, we show decreased levels of GABAergic synthesis enzymes and GABA_A_ receptors, and alterations in GABA transporters in AD rodent models. By contrast, there were no changes in GABA levels and GABA_B_ receptors. Our results suggest that the AD rodent models recapitulate the GABAergic system vulnerability seen in human AD.